# Effect of Dietary Supplementation of Probiotic *Aspergillus niger* on Performance and Cecal Microbiota in Hy-Line W-36 Laying Hens

**DOI:** 10.3390/ani12182406

**Published:** 2022-09-14

**Authors:** Milan K. Sharma, Dima L. White, Amit K. Singh, Haijun Liu, Zhigang Tan, Xianfeng Peng, Woo K. Kim

**Affiliations:** 1 Department of Poultry Science, University of Georgia, Athens, GA 30602, USA; 2 Guangzhou Insighter Biotechnology Co., Ltd., Guangzhou 510664, China

**Keywords:** probiotics, *Aspergillus niger*, egg production, egg quality, *Salmonella* spp., *Clostridium perfringens*, *Escherichia coli*

## Abstract

**Simple Summary:**

The use of antibiotics as a growth promoter in food-producing animals has been restricted throughout the world. As a result, many antibiotic alternatives have been evaluated in the poultry industry. *Aspergillus niger* is one of the probiotics of fungal origin that has shown promising effects in broiler production; however, few studies have been conducted on laying hens. Therefore, this study investigated the role of *Aspergillus niger* on performance, egg quality, and cecal microbial load of pathogenic bacteria in laying hens. The results showed that supplementation of Probioist^®^, a product containing the probiotic *Aspergillus niger,* at a rate of 220 mg/kg of diet, improved egg production and Haugh unit, and numerically decreased the cecal microbial load of *Clostridium perfringens*, *Salmonella* spp., and *Escherichia coli*.

**Abstract:**

This study aimed to investigate the role of the probiotic *Aspergillus niger* on the production performance, egg quality, and cecal microbial load of *Clostridium perfringens*, *Salmonella* spp., and *Escherichia coli* in Hy-Line W-36 laying hens. A total of 72, 45-week-old Hy-Line W-36 laying hens were randomly allocated to one of the three dietary treatments with six replicates, and each replicate had four individually caged laying hens (*n* = 6 and 4 hens/replicate). The hens in each treatment group were fed a corn and soybean meal diet (**Control**), a diet supplemented with bacitracin methylene disalicylate (**BMD**) at a rate of 495 mg/kg of feed (**Positive Control**), or a diet supplemented with *Aspergillus niger* (Probioist^®^) at a rate of 220 mg/kg of feed (**Probiotic**). Supplementing probiotics in the laying hen diet significantly increased egg production at weeks 3 and 6 compared with the Positive Control. Haugh unit, a measure of egg quality, was significantly higher in laying hens fed the probiotic diet compared with the Control or Positive Control at week 10. Furthermore, the Probiotic group had numerically lower cecal microbial loads of pathogenic bacteria (*Clostridium perfringens*, *Salmonella* spp., and *Escherichia coli*) compared with the Control and Positive Control groups. The results suggest that *Aspergillus niger* could be used as a probiotic to improve laying hen performance and egg quality.

## 1. Introduction

Over the past few decades, the poultry industry has undergone tremendous change to meet the increasing demand for inexpensive and high-quality proteins, such as meat and eggs [1]. Due to the continuous integration of various disciplines, including genetics, nutrition, and management, the poultry industry has become the fastest growing agricultural subsector, providing inexpensive sustenance to the growing global population [1,2,3]. Previously, antibiotics had been extensively used in the poultry industry as growth promoters to boost productivity to fulfill the growing demand for meat and eggs [4,5]. It has been observed that the inclusion of subtherapeutic dosages of antibiotics as a growth promoter in poultry diet improved the growth, production, and feed efficiency by reducing the pathogens (*Clostridium* spp., *Salmonella* spp., *Escherichia coli*, etc.) in the gastrointestinal tract and enhancing gut health [5,6,7,8]. Although the inclusion of subtherapeutic dosages of antibiotics in poultry diets has some beneficial effects, its use has been questioned, mostly due to the antimicrobial resistance among zoonotic microorganisms and antibiotic residues in the final products [1,3,9]. The use of antibiotics as growth promoters in food-producing animals is under great scrutiny in the U.S. The U.S. Food and Drug Administration (FDA) has called for banning the use of such antibiotics in food-producing animals [10]. With such restrictions on antibiotics as growth promoters in poultry, the search for effective antibiotic alternatives has increased over the past decades. 

The use of probiotics as alternatives to antibiotics has been studied multiple times over the past years, showing promising results [7,11,12,13,14]. Probiotics are living nonpathogenic microorganisms that, when supplied in adequate amounts, have beneficial effects on the host by altering the gut microflora and reducing pathogenic bacteria colonization in the gastrointestinal tract. This is accomplished by the competitive exclusion of pathogenic bacteria and enhancing the growth of beneficial bacteria [5,12,13]. Furthermore, their metabolites, such as short-chain organic fatty acids, hydrogen peroxide, or other metabolites, possess antimicrobial activity that prevents pathogenic bacterial growth [5,12,13]. Probiotics, when used as feed supplements, have beneficial effects on poultry health (improvement in gut health, immunomodulatory effect, and exclusion of pathogenic bacteria or protozoa) and performance, including body weight gain, feed intake, and feed conversion ratio [5,7,13]. *Lactobacillus*, *Bacillus*, *Enterococcus*, and *Bifidobacterium* species are among the most studied probiotics of bacterial origin; however, limited studies have focused on probiotics of fungal origin [13,15,16].

*Aspergillus niger* is a fungus belonging to the genus *Aspergillus* that has shown probiotic properties. It is the most common fungus in the environment and can be easily cultivated in laboratory conditions [17]. It is a good source of several bioactive compounds (citric, gluconic, and itaconic acids) and enzymes (α-amylase, proteases, cellulase, xylanase, L-asparaginase, α-galactosidase, phytase, and tannase), which are beneficial to poultry and may provide economic gain to the poultry industry [17,18,19]. *Aspergillus* spp. were found to be predominant in the gastrointestinal tract and cecum of poultry, indicating their viability and usefulness as a probiotic [20,21]. *Aspergillus niger* in poultry feed are beneficial as live fungi, which act as probiotics, whereas the dead cells can be used as prebiotics, supporting the growth of beneficial bacteria. Bioactive compounds produced by these fungi may help reduce the pathogenic bacterial population, and the enzymes enhance the digestion of carbohydrates and proteins [18]. In previous studies, 1.25% *Aspergillus niger* supplementation improved gastrointestinal health and reduced cecal coliform counts (*Salmonella* spp. and *Escherichia coli*) [12,22]. In addition, supplementing *Aspergillus niger* in the diet increased body weight gain, decreased feed intake, improved feed conversion ratio, and decreased abdominal and breast fat deposition in broilers [14,18]. Previously, it has also been observed that diets containing mixed probiotics that included *Aspergillus niger* improved egg production and quality [23,24,25,26,27]. Most of the studies mentioned above considered *Aspergillus niger* along with other probiotics, and these probiotics have been proven to have some benefits even when used alone. However, the effectiveness of *Aspergillus niger* as the only probiotic in Hy-Line W-36 commercial laying hens and its potential as a probiotic have not been explored. Therefore, in this study, we aimed to evaluate the effect of dietary supplementation of *Aspergillus niger* (Probioist^®^) on egg production, egg quality, and cecal microbial load of *Clostridium perfringens*, *Salmonella* spp., and *Escherichia coli* in Hy-Line W-36 laying hens. 

## 2. Materials and Methods

The experiment was conducted at the Poultry Research Unit at the University of Georgia, Athens, Georgia, and was approved by the Institutional Animal Care and Use Committee of the University of Georgia.

### 2.1. Experimental Design and Dietary Treatments

A total of 72 commercial Hy-Line W-36 white laying hens from a 45-week flock were placed into individual cages (50 cm length × 35 cm width × 40 cm height) in a temperature-controlled house where the temperature was set at 21 °C and the humidity at 40%. Each cage was equipped with a nipple drinker and a trough feeder. The birds were then allowed to adapt for two weeks. When the hens were 47 weeks old, they were weighed, and hens in similar body weight ranges were randomly allocated to three groups. The three groups were then assigned to three dietary treatments: (i) a corn and soybean meal control diet (**Control**); (ii) a diet supplemented with antibiotic bacitracin methylene disalicylate (**BMD**) at the rate of 495 mg/kg of feed **(Positive Control);** and (iii) a diet supplemented with the probiotic *Aspergillus niger* (Probioist^®^; Insighter Biotechnology, Guangzhou, China) at the rate of 220 mg/kg of feed (**Probiotic**). The main bioactive components of Probioist^®^ are *Aspergillus niger* (>5.0 × 10^8^ CFU/g) and its cultures, including live or dead fungi, and their metabolites, which are resistant to low pH. BMD at the rate of 495 mg/kg of feed and Probioist^®^ at the rate of 220 mg/kg of feed were added on top of the control diet to make the Positive Control and Probiotic diet groups, respectively. The diet formulation is shown in Table 1. Each treatment consisted of 6 replicates, each with 4 individually caged laying hens (*n* = 6 and 4 hens/reps). During the 10-week experimental period (47–57 weeks of age), ad libitum mash feed and water were provided with a 16:8 h light:dark period.

### 2.2. Layer Performance and Egg Quality

Egg production and mortality were recorded daily. Hen-day egg production (**HDEP**) was calculated every week as the number of eggs laid divided by the hen days during that period. Feed intake (**FI**; g/hen/day) and feed conversion ratio (**FCR**; kilogram of feed per dozen eggs) were calculated every three weeks. Feed offered was recorded for three weeks, and FI was measured by subtracting the remaining feed from the offered feed at the end of the third week. The HDEP, FI, and FCR were adjusted for mortalities throughout the early and late experimental periods. 

The external and internal qualities of the eggs were measured every three weeks. Three eggs were randomly collected per replicate to measure the external and internal qualities of the eggs. For the last sampling, three eggs were randomly selected over the course of two days, and egg quality was measured on the second day. To evaluate the external egg quality, the specific gravity, eggshell thickness, and eggshell percentage parameters were measured according to the previously described procedures [3,28]. Albumen height, Haugh unit, yolk percentage, and albumen percentage were measured as previously described to measure the internal egg quality [3,28]. 

### 2.3. Enumeration of Cecal Clostridium perfringens, Salmonella spp., and Escherichia coli

*Clostridium perfringens*, *Salmonella* spp., and *Escherichia coli* counts were evaluated from the cecal contents of laying hens. One laying hen per replicate was euthanized at weeks 3, 6, and 10 of the experiment, and ceca were aseptically collected and placed into sterile stomacher bags. The ceca were then weighed, 15 mL of the buffered peptone water (HiMedia Laboratories, Mumbai, India) was added, and the samples in the bags were homogenized in a stomacher (Neutec Group Inc., Farmingdale, NY, USA) for 60 s. The obtained solutions were then serially diluted 10-fold. 

*Clostridium perfringens* were counted using the pour plate method using brain heart infusion agar (Oxoid, Thermo Scientific, Lenexa, KS, USA). We poured 1 mL of the dilution on sterile plates in duplicate, and the media were poured and mixed by rotating in a figure-eight pattern. The plates were then incubated in an anaerobic chamber (Bactron Anaerobic Chamber, Sheldon Manufacturing, Cornelius, OR, USA) at 37 °C for 18 h. After incubation, visible black colonies were counted. The spread plate method was used to count the *Salmonella* spp. Using XLT4 agar (HiMedia Laboratories; Mumbai, India). Briefly, 100 µL of the serial dilutions was spread onto each respective agar plate in duplicate and was incubated at 37 °C for 24 h. The *Escherichia coli* were enumerated using 3M™ Petrifilm™ in duplicate (3M, Saint Paul, MN, USA). We poured 1 mL of the dilution on the petrifilm and was incubated at 37 °C for 24 h, and the colonies surrounded by the air bubble were counted as Escherichia coli colonies. Microbiological data are expressed as a logarithm of colony-forming units per gram of cecal weight (log CFU/g).

### 2.4. Statistical Analyses 

The data collected for performance, egg quality, and microbiology were analyzed using one-way analysis of variance (ANOVA) under a completely randomized design by the PROC GLM procedure of SAS 9.4 (SAS Institute Inc., Cary, NC, USA). The microbiology data were log-transformed prior to the analysis. At a *p*-value of ≤ 0.05, the means were considered statistically different, and the significance of treatment means was separated using Tukey’s HSD.

## 3. Results 

The average body weight of the laying hens was not significantly different either at the beginning or end of the experiment (*p* > 0.05). The effect of supplementing different dietary treatments (Control, Positive Control, and Probiotic) on egg production is shown in Table 2. Laying hens fed the diet supplemented with *Aspergillus niger* had higher HDEP during the periods of weeks 1–3, 1–5 (*p* = 0.003), 4–6 (*p* = 0.0028), and 1–10 (*p* = 0.003) compared with those in the Positive Control group. The *Aspergillus niger* (Probiotic) group had higher egg production (91.67%) than the Control (85.71%) and Positive Control (80.36%) groups at week 2 (*p* = 0.031). At week 6, the HDEP was significantly higher in the Control (92.86%) and Probiotic groups (91.43%) than in the Positive Control group (76.98%: *p* = 0.025). In addition, supplementation of the probiotic *Aspergillus niger* in the diet numerically increased egg production on weeks 1, 3, 5, and 10 compared with that in the Control and Positive Control groups. 

Supplementation of *Aspergillus niger* did not affect the egg weight, specific gravity, yolk percentage, albumen percentage, eggshell thickness, or eggshell percentage (Table 3; *p* > 0.05). However, egg weight was numerically higher in the Probiotic group than in the Control and Positive Control groups at week 6 (*p* = 0.418) and 10 (*p* = 0.097). Haugh unit was significantly higher in the Probiotic group (79.17) than in the Control (74.42) and Positive Control (75.00) groups at week 10 (*p* < 0.05). 

There were no significant differences among the Control, Positive Control, and Probiotic groups for FI and FCR at weeks 3 and 6 (Table 4; *p* > 0.05). However, FCR was numerically lower in the Control (2.00 kg feed/dozen eggs) and Probiotic groups (1.98 kg feed/dozen eggs) than in the Positive Control group (2.14 kg feed/dozen eggs) during weeks 0–3. Similarly, FCR was numerically lower in the Probiotic (1.53 kg of feed/dozen of eggs) and Control groups (1.48 kg feed/dozen eggs) than in the Positive Control group (1.61 kg/Dozen eggs) from 3–6 weeks of age. At week 10, FI was significantly lower in the Positive Control group (103.4 g) than in the Probiotic (113.1 g) and Control groups (116.4 g: *p* = 0.023). 

There was no significant difference among the treatment groups for *Escherichia coli*, *Salmonella* spp., or *Clostridium* spp. counts (Table 5; *p* > 0.05). However, supplementing probiotic *Aspergillus niger* at the rate of 220 mg/kg of feed numerically decreased the cecal bacterial counts for *Escherichia coli*, *Salmonella* spp. and *Clostridium perfringens* compared with both controls at weeks 3, 6, and 10. 

## 4. Discussion

Although the inclusion of subtherapeutic doses of antibiotics in poultry diets has some beneficial effects, the extensive usage of antibiotics has increased the safety risks associated with antimicrobial residuals in final products such as meat and eggs. Moreover, the development of antimicrobial resistance in zoonotic microorganisms is posing a great threat to global human health [10]. As a result, several countries have banned the use of antibiotics in animal production as growth promoters. It has been proven that using probiotics in poultry production has the same benefits as antibiotics, thus replacing the use of antibiotics in animal production. The use of the probiotic *Aspergillus niger* in broiler production and its effect on bird performance have been studied; however, minimal information is available on laying hens [15,20].

Supplementation with *Aspergillus niger* improved egg production in laying hens compared with the Control and Positive Control groups. This result is in accordance with those of previous studies where supplementing probiotics mixes containing *Aspergillus oryzae* improved egg production in laying hens [18,23,24,25,26,27]. In the current study, improved egg production in the Probiotic group may have been due to an increase in feed efficiency, which may have been primarily induced by the production of enzymes such as cellulase, xylanase, α-amylase, proteases, and α-galactosidase [17,18]. These enzymes produced by *Aspergillus niger* degrade the soluble nonstarch polysaccharides, cellulose, and nondigestible proteins, thus improving the nutritional value of the diet. In addition, it was observed that *Aspergillus* spp. produce enzymes that degrade the trypsin inhibitor in the soybean meal, leading to improved performance [19]. The presence of pathogenic bacteria in the gastrointestinal tract may affect nutrient digestion, absorption, and use. It was observed that the mannan-oligosaccharides from the yeast cell wall are capable of reducing the pathogenic bacteria in the GI tract, leading to the efficient diversion of nutrients from maintenance to production, eventually improving egg production [25]. The increase in egg weight could be attributed to the availability of excess nutrients in response to the enzymes produced by *Aspergillus niger* [19]. Improvement in the Haugh unit at week 10 could have be due to the antibacterial and antioxidant properties of the tannins released by the tannase produced by *Aspergillus niger*, improving reproductive health and thus enhancing the albumen height and Haugh unit [17,29,30].

Although nonsignificant, FCR was lower in the Probiotic group than in the Positive Control group for the first 6 weeks. This result is in agreement with those of previous studies, reporting that supplementation of probiotics did not produce significant differences in FI and FCR [23]. In contrast, others observed significantly decreased FI and FCR with the supplementation of probiotic *Aspergillus awamori or Aspergillus oryzae* [18,24,25,26]. In the present study, the reduced FI in laying hens fed a diet supplemented with antibiotics (Positive Control) may have caused the lower egg production in that group. The lower FCR in laying hens fed the diet supplemented with *Aspergillus niger* at weeks 3 and 6 may have be due to the availability of surplus nutrients in response to the potential action of *Aspergillus niger* enzymes (cellulase, xylanase, α-amylase, proteases, α-galactosidase, and phytase) [17,19]. These enzymes degrade the soluble nonstarch polysaccharides, cellulose, and nondigestible proteins, thus improving nutrient digestibility and utilization and reducing the FCR.

The gastrointestinal microbial population plays a vital role in maintaining gut health and normal digestive processes, influencing the overall performance of birds [31]. The decrease in the microbial load of *Escherichia coli*, *Salmonella* spp., and *Clostridium perfringens* in the current experiment is in agreement with that in a previous study reporting that supplementing *Aspergillus niger* at rates of 1% or 1.25% significantly reduced the *Escherichia coli* and increased the beneficial bacteria (*Bifidobacterium* and *Lactobacillus*) in the ceca [22]. The reduction in the bacterial load of *Escherichia coli*, *Salmonella* spp., and *Clostridium perfringens* in ceca may have been due to the competitive exclusion of pathogens by modulating the gut microflora and fortification of the gastrointestinal barriers by increasing the beneficial bacteria [12,22]. In addition, bioactive compounds produced by *Aspergillus niger* would be expected to act as antibacterial agents to selectively reduce the pathogenic bacteria in the ceca [29]. It was also postulated that *Aspergillus* spp. can create anaerobic conditions and provide a favorable environment for the growth and proliferation of *Bacillus subtilis*, *Lactobacillus plantarum*, and *Lactobacillus acidophilus*, and, in turn, the competitive exclusion of pathogenic bacteria [32]. These small changes in the microbial population of harmful bacteria in the current study may have played a vital role in bird performance, especially with increased egg production, because of the complex microbial interactions [31].

## 5. Conclusions

In conclusion, the dietary inclusion of Probioist^®^ (220 mg/kg), a product containing the probiotic *Aspergillus niger*, improved egg production and Haugh unit, and lowered the pathogenic bacterial load in the ceca of Hy-Line W-36 laying hens. Our findings show that *Aspergillus niger* possesses positive attributes for improving performance and reducing the pathogenic bacterial load and can be used as a probiotic alternative to antibiotics in commercial Hy-Line W-36 laying hens. In addition, further studies are needed to explore and verify the possible mechanisms that may have been involved in improving laying hen performance.

## Figures and Tables

**Table 1:** Ingredients and nutrient composition of Hy-Line W-36 laying hen ^1^ control diet (dry matter basis).

Ingredient	Amount (%)
Corn	58.80
Soybean meal (48% CP)	22.00
Limestone	10.45
Distiller’s dried grains with soluble	5.00
Dicalcium phosphate	1.57
Soybean oil	1.46
Salt	0.37
DL-Methionine	0.18
Lysine HCL	0.04
Mineral Preix ^2^	0.08
Vitamin premix ^3^	0.05
Calculated composition	Required/Supplied
ME (kcal/kg)	2800/2800
Crude protein (%)	16.00/16.24
Total Calcium (%)	4.40/4.40
Calcium particle size (fine: coarse, %)	40:60
Available P (%)	0.44/0.44
dLys (%)	0.74/0.78
dMet (%)	0.37/0.44
dTSAA (%)	0.67/0.67
dThr (%)	0.52/0.56

^1^ Control: a corn and soybean meal diet; Positive Control: antibiotic bacitracin methylene disalicylate at the rate of 495 mg/kg on top of the control diet; and Probiotic: supplemented with probiotic *Aspergillus niger* (Probioist^®^) at the rate of 220 mg/kg of feed on top of the control diet; ^2^ Mineral mix provided the following in g/100 g diet: Ca(H_2_PO_4_)_2_·H_2_O, 3.62; CaCO_3_, 1.48; KH_2_PO_4_,1.00; Na_2_SeO_4_, 0.0002; MnSO_4_·H_2_O, 0.035; FeSO_4_·7H_2_O, 0.05; MgSO_4_·7H_2_O, 0.62; KIO_3_, 0.001; NaCl, 0.60; CuSO_4_·5H_2_O, 0.008; ZnCO_3_, 0.015; CoCl_2_·6H_2_O, 0.00032; NaMoO_4_·2H_2_O, 0.0011; KCl, 0.10; dextrose, 0.40; ^3^ vitamin mix provided the following in mg/100 g diet: thiamine-HCl, 1.5; riboflavin 1.5; nicotinic acid amide 15; folic acid 7.5; pyridoxine-HCl, 1.2; d-biotin 3; vitamin B-12 (source concentration, 0.1%) 2; d-calcium pantothenate4; menadione sodium bisulfite, 1.98; α-tocopherol acetate (source, 500,000 IU/g), 22.8; cholecalciferol (source, 5,000,000 IU/g) 0.09; retinyl palmitate (source, 500,000 IU/g), 2.8; ethoxyquin, 13.34; I-inositol, 2.5; dextrose, 762.2.

**Table 2:** Effect of different dietary treatments on hen day egg production (%).

Treatment ^1^	Control	Positive Control	Probiotic	SEM	*p*-Value
Week 1	85.72	82.14	89.29	2.95	0.298
Week 2	85.71 ^ab^	80.36 ^b^	91.67 ^a^	2.68	0.031
Week 3	88.1	84.52	93.46	3.09	0.156
Week 4	93.65	83.33	90.48	3.65	0.751
Week 5	84.13	79.37	86.67	4.72	0.556
Week 6	92.86 ^a^	76.98 ^b^	91.43 ^a^	4.05	0.025
Week 7	88.1	92.86	90.47	3.83	0.694
Week 8	88.57	85.71	88.1	3.28	0.814
Week 9	92.86	89.29	92.86	4.22	0.784
Week 10	87.5	88.33	88.89	3.51	0.959
Week 1–5	87.46 ^a^	81.94 ^b^	91.07 ^a^	1.51	0.0003
Week 6–10	89.92	86.38	90.31	1.71	0.2115
Week 1–10	88.65 ^a^	84.08 ^b^	90.67 ^a^	1.15	0.0003
Week 1–3	86.51 ^b^	82.35 ^b^	91.74 ^a^	1.62	0.0009
Week 4–6	90.21 ^a^	79.89 ^b^	90.48 ^a^	2.41	0.0028
Week 7–10	89.12	89.06	90.08	1.79	0.9002

^a^^,^^b^ values within columns not sharing superscripts are significantly different at *p* < 0.05; ^1^ Control: corn and soybean meal diet; Positive Control: antibiotic bacitracin methylene disalicylate (BMD) at the rate of 495 mg/kg on top of the control diet; Probiotic: supplemented with probiotic *Aspergillus niger* (Probioist^®^) at the rate of 220 mg/kg of feed on top of the control diet.

**Table 3:** Effect of different dietary treatments on external and internal egg quality parameters.

Item ^1^	Egg Weight (g)	Specific Gravity	Yolk Percentage (%)	Albumin Percentage (%)	Haugh Unit	Shell Thickness (mm)	Eggshell Percentage (%)
Week 3							
Control	63.80	1.090	27.26	63.10	80.80	0.380	9.54
Positive Control	62.19	1.091	27.79	62.54	81.32	0.373	9.56
Probiotic	63.75	1.092	27.30	63.19	79.71	0.375	9.50
SEM	1.17	0.002	0.69	0.74	1.31	0.006	0.11
*p*-Value	0.553	0.400	0.844	0.813	0.680	0.735	0.933
Week 6							
Control	63.89	1.083	27.59	62.79	74.97	0.380	9.63
Positive Control	63.31	1.083	27.48	63.29	75.24	0.375	9.23
Probiotic	65.83	1.083	27.32	63.27	74.01	0.389	9.59
SEM	1.37	0.002	0.578	0.60	1.44	0.007	0.16
*p*-Value	0.418	1.000	0.947	0.796	0.829	0.382	0.174
Week 10							
Control	64.35	1.088	28.24	62.16	74.42 ^b^	0.392	9.60
Positive Control	63.49	1.088	26.93	63.73	75.00 ^b^	0.372	9.35
Probiotic	67.24	1.088	26.73	64.13	79.17 ^a^	0.383	9.72
SEM	1.19	0.002	0.79	0.92	0.89	0.010	0.24
*p*-Value	0.097	1.000	0.368	0.306	0.005	0.412	0.545

^a,b^ values within columns not sharing superscripts are significantly different at *p* < 0.05; ^1^ Control: corn and soybean meal diet; Positive Control: antibiotic bacitracin methylene disalicylate (BMD) at the rate of 495 mg/kg on top of the control diet; Probiotic: supplemented with probiotic *Aspergillus niger* (Probioist^®^) at the rate of 220 mg/kg of feed on top of the control diet.

**Table 4:** Effect of different dietary treatments on feed intake and feed conversion ratio.

Item ^1^	FI (g/d/Bird)	FCR (kg Feed/Dozen Eggs)
Week 3		
Control	119.2	2.00
Positive Control	117.7	2.14
Probiotic	119.8	1.98
SEM	2.34	0.07
*p*-Value	0.814	0.282
Week 6		
Control	110.9	1.48
Positive Control	106.1	1.61
Probiotic	110.0	1.53
SEM	5.02	0.060
*p*-Value	0.775	0.334
Week 10		
Control	116.4 ^a^	1.59
Positive Control	103.4 ^b^	1.59
Probiotic	113.1 ^a^	1.59
SEM	2.94	0.14
*p*-Value	0.023	1.000

^a,b^ values within columns not sharing superscripts are significantly different at *p* < 0.05; ^1^ Control: corn and soybean meal diet; Positive Control: antibiotic bacitracin methylene (BMD) disalicylate at the rate of 495 mg/kg on top of the control diet; Probiotic: supplemented with probiotic *Aspergillus niger* (Probioist^®^) at the rate of 220 mg/kg of feed on top of the control diet.

**Table 5:** Effect of different dietary treatments on microbial load of *Escherichia coli, Salmonella* spp., and *Clostridium perfringens* in the ceca.

Item ^1^	*Escherichia coli* (Log CFU/g)	*Salmonella* spp. (Log CFU/g)	*Clostridium perfringens* (Log CFU/g)
Week 3			
Control	6.03	6.28	6.32
Positive Control	6.39	6.08	6.11
Probiotic	5.62	5.52	5.40
SEM	0.35	0.42	0.32
*p*-Value	0.342	0.445	0.168
Week 6			
Control	6.96	5.92	4.51
Positive Control	6.73	5.78	3.86
Probiotic	6.76	5.62	3.96
SEM	0.26	0.28	0.23
*p*-Value	0.798	0.734	0.122
Week 10			
Control	6.76	5.47	4.65
Positive Control	5.88	4.62	4.04
Probiotic	5.67	4.65	3.41
SEM	0.49	0.50	0.39
*p*-Value	0.286	0.429	0.123

^1^ Control: corn and soybean meal diet; Positive Control: antibiotic bacitracin methylene disalicylate (BMD) at the rate of 495 mg/kg on top of the control diet; Probiotic: supplemented with probiotic *Aspergillus niger* (Probioist^®^) at the rate of 220 mg/kg of feed on top of the control diet.

## Data Availability

The data presented in this study are available in this paper.
